# First Case Report of a Sporadic Adrenocortical Carcinoma With Gastric Metastasis and a Synchronous Gastrointestinal Stromal Tumor of the Stomach

**DOI:** 10.1097/MD.0000000000001549

**Published:** 2015-09-18

**Authors:** Attila Kovecsi, Ioan Jung, Tivadar Bara, Tivadar jr. Bara, Leonard Azamfirei, Zsolt Kovacs, Simona Gurzu

**Affiliations:** From the Department of Pathology (AK, IJ, ZK, SG); Department of Surgery (TB, TjB); and Intensive Care Unit, University of Medicine and Pharmacy of Tirgu-Mures, Targu Mures, Romania (LA).

## Abstract

Adrenocortical carcinoma is a rare tumor with high aggresivity that can associate systemic metastases.

A 71-year-old man was hospitalized for gastric cancer. The abdominal computed tomography also revealed a tumor above the right kidney. Total gastrectomy and right adrenalectomy were performed. The encapsulated tumor of the adrenal gland weighed 560 grams and presented diffuse tumor architecture under microscope, with capsular, sinusoidal, and vascular invasion. The large tumor cells had a polygonal shape, with slight basophilic, eosinophilic, or vacuolated cytoplasm, pleomorphic nuclei, and a high mitotic rate. In the stomach, the protruded tumor was covered by normal mucosa; under microscope, the tumor cells were observed only in the submucosal layer. In primary adrenal tumor and gastric metastasis the tumor cells were marked by vimentin, inhibin, synaptophysin, neuron-specific enolase, and calretinin. Based on these criteria, the diagnosis of adrenocortical carcinoma (ACC) with gastric metastasis and no lymph node metastases was established. A synchronous 10 × 10-mm-sized gastrointestinal stromal tumor (GIST) of the stomach, without mitoses, was also identified.

So far, as we know, this is the 15th case of ever reported synchronous/metachronous sporadic ACCs; the ACC-related gastric metastases either synchronous ACC and GIST, has not been reported in the literature previously.

## INTRODUCTION

Adrenocortical carcinoma (ACCs) is a rare tumor with an incidence of 0.02–0.2% of all malignancies; the reported annual incidence being about 0.7–2 cases per million people.^1–5^ It can involve both children and adults, the median age being about 56 years (range 1–91 years).^1^ ACC can be endocrinologically active or nonfunctional tumor, being diagnosed incidentally, because of loco-regional compression phenomenon or based on the distant metastases. The lymph node metastases are very rare, but the systemic ones are present in about half of the patients.^1,5^ This is the reason, despite the postoperative adjuvant therapy; it is extremely aggressive, the overall 5-year survival rate of resectable metastatic tumors being below 30%.^1^

The differential diagnosis between primary ACC, pheochromocytoma, and a metastatic tumor is difficult to be performed, it being based on a proper correlation between clinicopathological and immunohistochemical aspects.^5,6^

In this article, we present a case of a nonfunctional sporadic adrenocortical carcinoma with a gastric metastasis masquerading as a primary gastric cancer. To date, no cases presenting this metastatic localization were reported. The second particularity of the case is its synchronous association with a gastrointestinal stromal tumor (GIST) of the stomach, incidentally diagnosed at the macroscopical examination of the surgical specimens. To our best knowledge, this is the first report in PubMed-citated English literature about coexistence of GIST with ACC and the 15th one that presents a synchronous tumor associated with a sporadic ACC. Considering these particularities, we present the clinical, histopathological, and immunohistochemical criteria used for its diagnosis and also a pertinent review of the literature about metastatic behavior of ACC and its synchronous or metachronous tumors.

## CASE PRESENTATION

**FIGURE 1:**
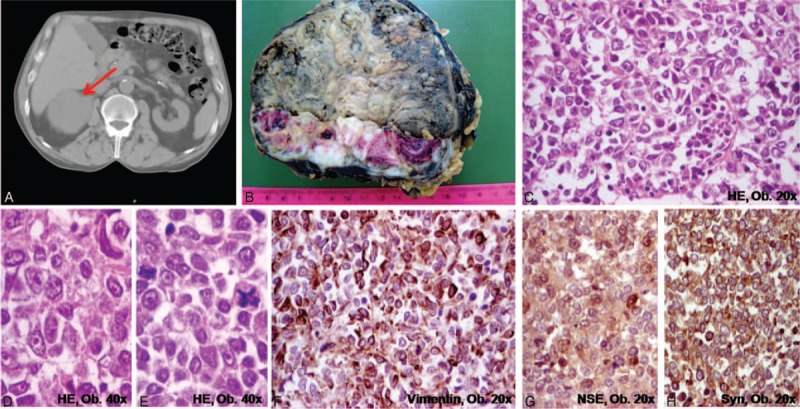
Adrenocortical carcinoma of the right adrenal gland. A, CT-scan; B, Macroscopic aspect. C–H, Microscopically, the clusters of polygonal-shaped cells with eosinophilic cytoplasm, well-defined nucleoli, and atypical mitoses (C–E) are marked by vimentin (F), neuron specific enolase (G), and synaptophysin (H).

A 71-year-old previously healthy man was admitted to our hospital with moderate weight loss (5 kg in 2 mo), epigastric pain, vomiting, and fatigue. A gastric biopsy was performed 2 weeks before in another hospital, and the diagnosis was undifferentiated gastric carcinoma. On physical examination, normal bowel sounds and soft, nontender abdomen were observed. The upper gastrointestinal endoscopy confirmed the gastric tumor that protruded in the gastric body. Also, the computed axial tomography scan revealed a tumor mass above the right kidney (Figure 1) and excluded liver and pulmonary metastases; the left adrenal gland was in normal limits. No personal or familial history of cancer or endocrine disorders was noted.

The laboratory examinations showed slight anemia (hemoglobin 10.6 g/dl, hematocrit 31%) and slight elevated serum level of transaminases (aspartate transaminase [AST]: 88 U/L and alanine transaminase [ALT]: 137 U/L). The renal function (creatinine 1.23 mg/dL, urea 40 mg/dL) and the serum cortisol level were normal; glycemia and amilasemia were in normal limits (79 mg/dL and 64 U/L, respectively). A moderate hypertension (175/95 mm Hg) with rhythmic arterial pulse (92 beats per minute) was detected.

Laparotomy with total gastrectomy, omentectomy, D1 lymphadenectomy, splenectomy, and right adrenalectomy was decided. Signed informed consent was preoperatively obtained to perform surgery and publish the case details.

The first intraoperative step was the right adrenalectomy followed by gastrectomy. This was decided because gastric resection is considered a septic intraoperative step. The adrenal tumor was encapsulated, well defined, without renal invasion, and ipsilateral nephrectomy was not necessary. In the stomach, due to the fact that the tumor was localized in the gastric proximal third, corresponding to the gastric fundus, presented pancreatic adherences and a synchronous intraparietal nodule was palpated on the posterior gastric wall, at 60 mm from the main tumor, total gastrectomy and splenectomy, with end-to-side Roux-en-Y eso-jejunoanastomosis was performed. It is worth mentioning that, to assure an optimal outcome, such intervention can only be done by a surgical team with rich experience in adrenal masses resection, and that the operating time was about 3 hours. Although splenectomy is considered a negative prognostic factor, it was the optimal surgical approach, based on the proximity of the gastric tumor to the spleen and presence of the synchronous gastric tumor in the proximal stomach.

Macroscopic examination of the right adrenalectomy surgical specimen showed a 13 × 10 × 6- cm-sized encapsulated tumor weighing 560 grams. The surface was partially bosselated. On the cut section, the tumor had a nodular aspect, the grayish solid areas, with hemorrhages, and necrotic zones, being separated by fibrous septa (Figure 1). Focal areas with capsular invasion were seen.

**FIGURE 2:**
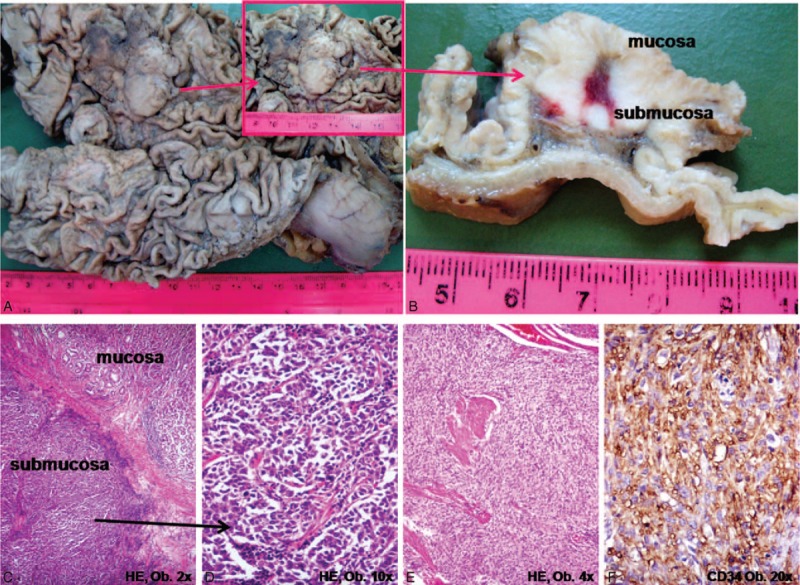
Gastric metastasis from adrenocortical carcinoma. A,B, Macroscopically, the protruded tumor is located in the gastric body, in the submucosa. C,D, Microscopic examination shows clusters of polygonal-shaped cells with eosinophilic cytoplasm with similar aspect with the primary tumor from Figure 1. E,F, The synchronous gastrointestinal stromal tumor (GIST) of the stomach displays CD34.

Gross examination of the stomach revealed a 2 × 1.7 × 1-cm-sized protruded tumor, covered by intact mucosa and localized on the posterior wall of the gastric body, corresponding to the gastric fundus. On a cut section, the white-grayish tumor was limited to the submucosa (Figure 2). On the external surface of the stomach, at 60 mm from, on the posterior gastric wall, another 10 × 10-mm-sized nodular tumor was identified, this being limited to the muscularis propria.

Histopathological examination of the adrenal tumor showed a well-vascularized tumor with a diffuse architecture, the tumor cells being separated by fibrous septa. Necrosis and hemorrhages were expanded; large inflammatory areas with bone metaplasia were also observed. At a high power view, we noticed large, polygonal-, or oval-shaped tumor cells, with slight basophilic, eosinophilic, or vacuolated cytoplasm, focally with intracytoplasmic hyaline globules. The vacuolated cells comprised less than 25% of the tumor. Large pleomorphic nuclei, with several nucleoli and a high mitotic rate (>20 mitoses/50 HPF), with multiple atypical mitoses, were also observed (Figure 1). The connective capsule presented tumor invasion; the tumor cells crossed the tumor capsule and invaded the pericapsular adipose tissue. Tumor invasion was also noticed in the sinusoid capillaries; large veins with huge tumor emboli and intravascular platelet conglutination were also present. The tumor displayed 9 of the 9 Weiss's criteria^3^ for malignancy: expanded areas of necrosis, capsular/vascular and sinusoidal invasion, high mitotic count, abundant atypical mitoses, high nuclear grade, diffuse architecture, and few vacuolar cells.

The microscopical examination of the stomach confirmed that the tumor was limited to the submucosal layer, without involvement of the gastric mucosa/muscularis mucosae/muscularis propria. The tumor cells presented a similar aspect with those from the adrenal tumor, without necrotic and/or hemorrhagic zones and without vascular invasion (Figure 2).

Immunohistochemically, the tumor cells from the adrenal and gastric submucosal tumor had the same characteristics. The tumor cells displayed diffuse positivity for vimentin, inhibin, synaptophysin, and neuron-specific enolase (NSE) (Figure 1). Focal expression of calretinin, keratin AE1/AE3, and epithelial membrane antigen (EMA) was also observed. Chromogranin, CD56, keratin 7, and 20, HMB45, and S100 were negative. The Ki67 proliferative index was approximately 30%.

The synchronous GIST was composed of spindle-shaped tumor cells, focally with epithelioid aspect and without mitoses that displayed diffuse positivity for CD34 and DOG-1 and focal expression for c-KIT, S100, and CD56, without smooth muscle actin (SMA) positivity (Figure 2).

Based on the macroscopical and microscopical features, as correlated with the clinical aspect and immunohistochemical profile, the final diagnosis was adrenocortical carcinoma with gastric metastasis and synchronous GIST of the stomach with very low malignant potential. The primary gastric carcinoma was excluded based on the tumor location (limited to the gastric submucosa) and the immunohistochemical profile. The patient died 1 year (12 months) after surgery without adjuvant therapy (patient's refusal). No necropsy was performed.

## DISCUSSION

ACC rarely associates lymphatic-borne metastases in the mediastinal or neck lymph nodes; however, the systemic metastases are frequent, being distributed throughout the body, without a specific spreading pathway.^1,6^ In most of the cases, the liver and lung are affected, followed by the bone and peritoneum.^1,6^ Other organs were also reported to be involved, such as ovaries, skin, brain, pancreas, spleen, and tongue.^1,6^ To our knowledge, no gastric metastases have been reported to date (March 2015). In our case, submucosal gastric metastasis was the reason of hospitalization, being confused with a primary gastric cancer. However, differential diagnosis between a primary ACC and a metastatic adrenal metastasis is very difficult to be performed and the immunohistochemical panel is not always helpful. In the present case, the focal keratin AE1/AE3 positivity increased the diagnostic difficulty, especially in the first endoscopically performed biopsy. The ACCs usually display diffuse positivity for inhibin, vimentin, and synaptofizin and are negative for chromogranin, EMA, and keratins,^2–12^ although EMA and keratin AE1/AE3 can present focal positivity,^6^ such as in this case. The tumor size and the optical microscopy using the usual staining hematoxylin and eosin constitute the basic criteria for differential diagnosis, the immunohistochemical panel being only used to confirm the diagnosis.

Only 14 cases with synchronous or metachronous tumors associated with sporadic ACC were reported to date, this being the 15th; the clinicopathological data of these cases are shown in Table 1.^2,3,7–17^ No GIST-associated ACCs have been reported before; only one 3-cm-sized right adrenal adenoma was incidentally found at ultrasound examination in a 48-year-old man with a gastric GIST.^18^ ACC can also be developed as part of the following inherited familial cancer syndromes: Li-Fraumeni syndrome, familial Becwith–Wiedemann syndrome, Gardner syndrome, and multiple endocrine neoplasia type 1.^2^

**TABLE 1:**
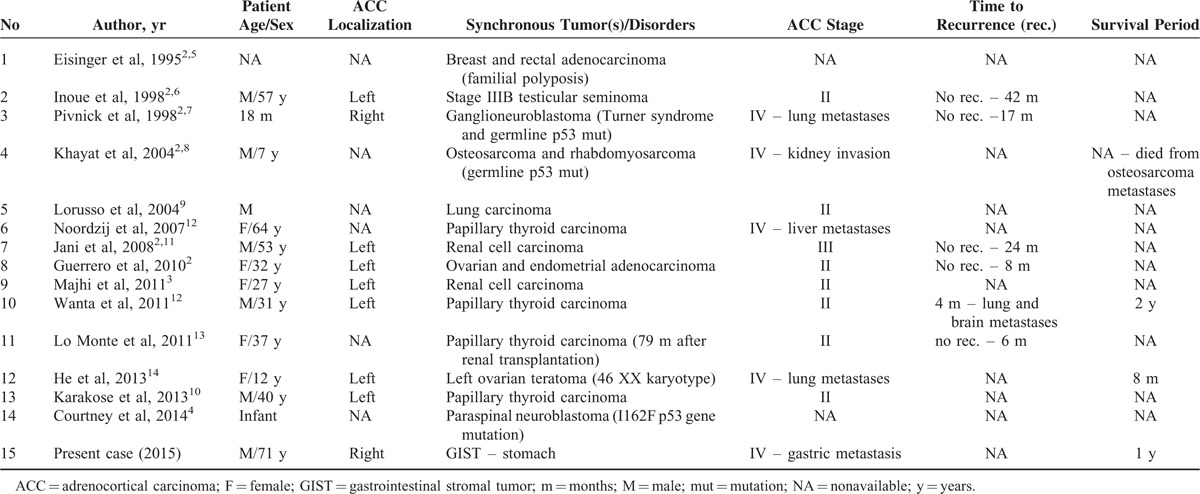
Clinicopathological Characteristics of Synchronous Tumors Diagnosed in Patients With Adrenocortical Carcinoma

Although screening programs would be useful for early detection of such cases, low incidence of ACC does not allow these programmes. Moreover, in organs such as stomach, a metastasis from ACC can be easily confused with a poorly cohesive gastric carcinoma, especially in biopsy specimens. At the same time, most of the patients with ACC present steroid hormone excess (40–60%) or abdominal mass effect (30%) but 15–20% of them are nonsymptomatic, the hematogenous-borne metastases being frequently the first clinical sign.^5,6^ Based on our experience in the field, we suggest performing of an abdominal computer tomography scan in any patient with gastric cancer, especially for cases with diffuse architecture on biopsic specimens detected in patients younger than 50 years, to detect the possible synchronous tumors, such as GIST or adrenocortical tumors, but also for an preoperative examination of the lymph node status and identification of the skip metastases in the distant lymph nodes.^19^ Regarding the ACC, for any adrenal mass, careful follow-up and surgical excision for any tumor larger than 4 cm and/or with necrosis, hemorrhage or calcification, should be performed to avoid malignization.^5^ The surgical excision can only be done by an experienced surgeon, which performs at least 10 adrenalectomy per year; the optimal surgical approach is one of the very important prognostic factors.^5^ The adrenalectomy can be performed through open surgery or laparoscopy but we recommend open surgery, especially for cases in which preoperative examination was not coherent and the surgeon does not have a rich experience in the field. Our observation is in line to some of the literature data that revealed a rate of peritoneal carcinomatosis from 25% of patients, after open surgery, to 60% after laparoscopic adrenalectomy, although the opinions are controversial.^5^ Nephrectomy is recommended only for cases with gross invasion of the unilateral kidney.

The present case highlights the importance of a correct and complex examination of patients to identify a possible synchronous tumor. In case of metastases with unknown origin, an ACC should be taken into account for differential diagnosis, especially in anaplastic carcinomas, independently from their location.
